# Drug development: successes, problems and pitfalls—the industry perspective

**DOI:** 10.1136/esmoopen-2016-000033

**Published:** 2016-01-27

**Authors:** Wolfgang Wein

**Affiliations:** Wein, Merck (KGaA), Vienna, Austria

**Keywords:** Drug development

## Issue 1: Demographics

Currently, the world population is heading for an 11 billion people peak in the next decades. Owing to the successful and victorious efforts and strategies of the academia, pharmaceutical industry and regulators, the average life expectancy of the European population will be close to a hundred years by the mid-century, because death rates of infectious or cardiovascular diseases as well as of many others have constantly declined. Haematological cancers today can be cured to a considerable extent or converted into well-controlled chronic states. Also, the incidences of a number of important solid cancers have declined, because of successful tobacco control, screening, prevention strategies or eradication of *Helicobacter pylori* infections. On the contrary, the longer the mean life expectancy of the population, the more likely will the occurrence of cancers become in the ageing population and significant challenges will arise in terms of defining the best treatment strategies for these age groups and to develop useful drugs for this population that are effective with a high tolerability. Today, the cut-off for the elderly in clinical trials is often 65 years, but what if by 2050 one-third of the population in EU countries will be above this age and require treatment? The other important consideration is, of course, cost of treatment, but since this is the only topic which we constantly get communicated again and again by several players, and it is not part of development in principle, I will refrain from comments and can only point to the study mentioned in the next item.

## Issue 2: ‘Explosion’ of molecular biology

Whoever attended ESMO in 1995 could find maybe only three or four new drugs, which were all chemical entities or synthetic versions of naturally occurring plants. Today, at every oncology meeting, the sheer enormity of new molecules, phase I, II and III trials, competitors and technologies makes it almost impossible to even memorise the most important advances. Even when focusing on just one tumour entity, it has become a true challenge to stay on top of the development or, even better, stay ahead of it in a leading position. This has become possible through the explosion of knowledge and technologies in molecular biology, which helps us to unravel the secrets of the cell and signalling pathways and to better understand the mechanisms behind certain tumour types, but at the same time, where we once knew one receptor, this one receptor has now been revealed to have 5 subreceptors, 2 resistance mechanisms, 10 downstream signalling pathways, etc, so much so that the complexity is ever growing.

This leads to several consequences: first, strategically, it becomes very difficult to plan your next studies and development programmes, because, owing to the complexity, there is a great deal of uncertainty about outcomes, new drugs arrive in daily practice, new findings might change the whole trend in a field, like the discovery of a new resistance mechanism, a new biomarker, etc. Given the biological complexity, the intelligent combination of preclinical and clinical studies has thus become standard, requiring a complementary rather than a strictly separated approach, even if the clinical development remains the mainstay of drug development. The full development cost for a new cancer drug has by far exceeded the US$2 billion mark. A recent study of the Tufts centre of the study of drug development by Di Masi and co-workers in 2014^1^ ^2^ shows that the out-of-pocket cost for one compound ranges around US$1.4 billion, while the fully loaded cost for a new drug to the company ranges at around US$2.6 billion today. The regulatory approvals have sharply declined from 1998 to 2008, moving back to the level of the early nineties by 2013. Of the 1442 compounds studied, 7% were approved, 80% had been discontinued and 13% were still active in development. This means that one will need to put 8.5 drugs in development in order to receive one approved drug, the average development time of which takes up to 11–12 years.

## Issue 3: Development time versus explosion of new findings

While we have become used to the speed of development cycles in telecommunication, where new devices appear almost in a quarterly fashion, we sometimes forget the pace of new findings in molecular biology versus the slow motion of drug development, which as described is around 12 years for a new drug in oncology, while the patients are waiting. Now, even if a company was very quick and successful in mounting a new clinical trial, even within the time to set it up, a new finding, a new biomarker or a new biological entity could have come up in the meantime, putting the whole trial in question. Since the classical design of phase III trials allows for one end point, for one major question only, on which the entire fate of the drug rests, there is an increasing discrepancy between the slow pace of development, the long development time and the massive stream of new findings, related questions and related new complexity to be answered. I have no recipe or solution for this issue, but something needs to happen in terms of trial methodology, statistical approaches, computer simulations or whatever to overcome this gap, because we waste a lot of time and burn enormous societal resources, which could be spent in a much more efficient way. Some considerable improvements on the regulatory side have happened or are on the way, like the *Accelerated Assessment* and *Conditional Marketing Authorization* procedures of the EMA with more flexible and expedited approaches and the *21st Century Cures Act*, including also patient preferences into the procedure, which is on its way in the USA. On the methodical side, some smarter approaches like *Adaptive Trial Design* or *Continuous Reassessment Method (CRM)* are being used increasingly in phase I. One major goal should be to shorten time to drug failure, so as to prove insufficient efficacy or too high toxicity early on in development, in order to terminate the project *before* it enters the expensive phase III phase. Still, phase III trials remain huge and are becoming always larger to meet statistical requirements.

## Issue 4: Statistical dilemma

While the ever increasing and improving efficacy in terms of overall survival is the landmark of progress in oncology, it comes with some interesting consequences in terms of classical statistical techniques. For instance, some 10 or 15 years ago, a 2-month increase in non-small cell lung cancer, let us say from 8 to 10 months overall survival (OS), was felt to be an important step forward and yielded the right increment in percentage to achieve statistical significance levels like p<0.001. A similar improvement in survival time in metastatic colorectal cancer of 2 months absolute, from, for example, 29 to 31 months of a new drug combination compared with an old one, will be regarded as a minor improvement and will not reach statistical significance for regulatory purposes. So these two absolute months of survival are seen as not very important, while the 2 months from 8 to 10 months OS were. This in turn puts pressure on the ability to differentiate between competitive approaches, which is very difficult to estimate, when after phase II one is just getting more familiar with the efficacy, toxicities dosing regimen and schedules of the new drug. Yet another major pitfall which comes into play is the influence of the increasing number and efficacy of second-line, third-line and fourth-line therapies, which are in the best interest of the patients, but in terms of crossover of treatment arms will compromise the results and could sometimes even help the weaker substance more than the more active one. The option of resorting to progression free survival (PFS) as a primary end point has been discussed intensively and might be ‘cleaner’ in a way, but will also bring new complexities in terms of accuracy in measuring time-to-event, influence of toxicities, early dropouts, radiological methods used, etc. Therefore, the number of patients per treatment arm will constantly increase and has grown from 100 to 250 patients per arm in the 90s up to 500 to 1000 patients in the newer generation of phase III trials, not to talk about non-inferiority studies.

## Issue 5: Biomarkers and whole genome sequencing

A fantastic achievement of recent years was the identification of certain specific tumour genomic alterations serving as biomarkers, allowing better understanding of the resistance patterns and hence to select drugs to which these tumours will respond or avoid drugs which are inactive. This has not yet led to personalising cancer care, but at least to detecting patient populations with a tumour carrying the specific pattern, which will respond to treatment. Even though the detection of biomarkers has resulted in a much more rational selection of therapies and biomarker testing has become routine for many tumour types, there are still issues in the day-to-day treatment situation, be it problems with the specimens, waiting and turnaround times, ignorance and lack of information conveyed to the patient. Also, although everybody talks positively about biomarkers, when it comes to reimbursement at the payor level, it may still take time and effort until a new biomarker is reimbursed, even though it is the best strategy any healthcare system could employ to prevent the use of expensive but inactive drugs by reimbursing a test for maybe €100. Biomarker testing, however, adds a whole range of new complexities to the pharmaceutical company's development programme when involving small diagnostic companies, which lack global presence or large-scale manufacturing experience, and new or more accurate biomarker tests, which appear during the course of a phase III trial, may cause issues of retrospective analysis, statistical power and size of patient groups detected by the more accurate or new test. Today, companion diagnostics need to be developed in parallel from the start of the programme and implemented in the hospital setting of many different countries, adding to the complexities.

Another significant step forward is the full sequencing of a tumour's entire genetic structure and thus to better understand changes within the tumour during treatment and to better select drugs which are most active in a certain tumour type as pioneered in a study published by Von Hoff and coworkers.^3^ What was once an incredibly expensive procedure has now come down to around €1000 and will possibly allow for an optimal selection of active drugs and also for monitoring of driver, passenger and backseat mutations and their influence of the tumour dynamics for further matching and selection of drug cocktails or sequences. A particularly powerful combination, and I would bet all my money on this one, will be the combination of genomics with artificial intelligence. By analysing the patterns of the tumour mutations, which are different in every tumour, and by entering the data of all individual mutation characteristics into huge databases, one can apply Bayesian statistics as used today in popular search machines, which might 1 day be able to recommend the optimal treatment scheme for a patient's individual tumour, like they are able today to predict your preferred next book to be ordered from Amazon. Cancer may not be defeated by humans, but it will be defeated eventually by the computer.

## Issue 6: Regulated Individual Early Access Programs

Owing to the slow pace of drug development in oncology and the pressing need of patients with cancer and their increasingly high level of information through the internet, there will be more and more situations where, for example, the ideal tyrosine kinase inhibitors (TKI) for a certain mutation is not yet available on the market but the patient is in need and could potentially benefit. For such situations, certain highly controlled individual *Early Access Programs* restricted to validated Comprehensive Cancer Centers only, including precise regulations and documentation as soon as randomised phase II data are reported, could be a win-win-win situation. It could provide the urgently needed medicine to patients much earlier, it would give oncologists and researchers experience with the new drugs early on, even if the phase III programme may run in other countries, and it could provide important signals about safety and efficacy in various tumours to the industry early on. Even though a trade-off in terms of risk–benefit will always be inherent, particularly in patients with late stage cancer, the benefits of such a programme may outweigh the potential risks.

## Issue 7: Larry Einhorn—‘Be careful about irrational exuberance, beware of irrational pessimism’

The greatest success of the recent years I have saved for the end, since the enormous progress of immuno-oncology is obvious and the field thriving. Even some 6 or 7 years ago, advocates of immuno-oncology were regarded as somewhat exotic or just crazy when investing in immuno-oncology projects. Today, everybody was the one who always knew that it was a great development to come, as it always goes in history. This is a really tremendous success, *however*, and the activation of the body's own immune system, after unmasking the cancer, has added a whole universe of opportunities to existing chemotherapy, biologics and TKIs. Even if we are just at the beginning of developing new immuno-oncology drugs, to establish biomarkers and combining and sequencing them among each other and with existing therapies, it has become very clear that this strategy is going to work and brings huge benefits. Nevertheless, every one of us sees family members, friends and patients die from cancer even though the best strategies are being applied. So I wanted to remember the famous quote of Larry Einhorn when one of the biggest hopes failed in the year 2000: ‘Be careful about irrational exuberance, beware of irrational pessimism’.
